# Results of the transition from posterolateral to anterior minimally invasive approach for total hip arthroplasty

**DOI:** 10.1186/s13018-023-04291-6

**Published:** 2023-10-31

**Authors:** Daniel Vernaza Obando, Kelly Johana Gallego, Sofía Gonzalez, Alejandro Gallego Álvarez, María Bautista, Alfredo Sánchez-Vergel

**Affiliations:** 1 Facultad de Medicina, Universidad Icesi, Cali, Colombia; 2 Centro de Investigaciones Clínicas, Fundación Valle del Lili, Cali, Colombia; 3 Servicio de Ortopedia y Traumatología, Hospital Universitario Fundación Valle del Lili, Carrera 98 # 18-49, Cali, 760032 Colombia

**Keywords:** Hip, Arthroplasty, Minimally invasive surgical procedures, Intraoperative complications, Postoperative complications

## Abstract

**Background:**

The anterior minimally invasive (AMI) approach reduces soft tissue damage, risk of dislocation and enhances recovery, but it is associated with certain complications. The aim of this study is to compare the outcomes of patients who underwent total hip arthroplasty (THA) through posterolateral (PL) and AMI approaches performed by the same surgeon, in order to determine the learning curve associated with this new approach.

**Methods:**

This retrospective cohort study included patients who underwent THA via PL and AMI approach between 2017 and 2022, with a minimum follow-up of 1 year. Hip fracture and oncologic patients were excluded. Demographic variables, functional scores and perioperative complications were assessed. A bivariate analysis was performed to identify differences between groups.

**Results:**

Data of 124 AMI and 120 PL patients were analyzed. Demographic characteristics among groups were homogeneous. Functional outcomes at 3 months were superior for AMI (Oxford: 43 vs. 38; *p* < 0.05), no dislocations were identified (0% vs. 4.2%; *p* < 0.05) and no differences in the transfusion rate were found (6.5% AMI vs. 6.7% PL; *p* = 0.996). Infection rate was 4% for AMI and 3.4% for PL (*p* = 0.572). Surgical time was shorter for the PL approach, but the median surgical time of the last 25 AMI cases was shorter.

**Conclusions:**

The AMI approach is an excellent alternative for patients requiring THA. Although surgical time and perioperative bleeding were greater during the learning curve, this approach offers improved functional outcomes and a lower dislocation rate, without significant differences in transfusion and infection outcomes, demonstrating that responsible innovation and safe implementation of new techniques is possible.

## Introduction

Hip osteoarthritis is a very common condition in the adult population, with a cumulative risk of 25% at 85 years old [1], and a risk of requiring a total hip arthroplasty (THA) of 10% after the age of 50 [2]. It is estimated that by the end of this decade, more than 500.000 THA will be performed annually in the USA [3, 4].

To achieve superior outcomes for this increasing population, a shift from the commonly used posterolateral (PL) approach [5] to the anterior minimally invasive (AMI) approach has been evidenced recently [6]. The AMI approach reduces soft tissue damage and offers numerous advantages over the PL, including less postoperative pain, early rehabilitation, and lower risk of dislocation [7–10]. Nevertheless, it has also been described that the limited exposure of this approach is associated with early implant loosening, periprosthetic fractures, and a higher rate of neurovascular injuries [11–14].

The risk of these complications is higher in less experienced surgeons [15] and it has been described that the learning curve for THA through the AMI approach is at least 50 procedures to obtain comparable results with the traditional approach [16–18]. Given that the increase in the use of the AMI approach has been debated [19], the implementation of new techniques and technologies to impact patients’ outcomes must be safe and accountable [20, 21].

The purpose of this study is to compare the clinical and functional outcomes of patients undergoing THA through the PL approach and the AMI approach during the period of transition of a single surgeon at an academic hospital.

## Methods

This retrospective cohort study included adult patients (≥ 18 years) undergoing primary THA via PL and AMI approach between January 2017 and May 2022, with at least 1-year follow-up. Patients with acute hip fractures and malignancy were excluded. All surgeries were performed by a single fellowship trained surgeon, first in PL and later in AMI approach by assisting experienced surgeons, cadaveric dissections and by being assisted by other experienced surgeons.

Sociodemographic variables, comorbidities, body mass index (BMI), ASA classification, indication for surgery, intraoperative and postoperative complications, length hospital stay, surgical site infection, mortality, functional outcomes in the Oxford Hip Score (OHS) and surgical time were assessed.

### Surgical technique

#### Anterior minimally invasive approach

The patient is positioned supine in the AMIS™ (Anterior Minimally Invasive Surgery) Mobile Leg Positioner design by Medacta (Medacta Corporate, Strada Regina, Switzerland). The skin incision is a line positioned 1 cm lateral and 1 cm distal to the anterior superior iliac spine, aimed distally toward the Gerdy’s tubercle. The fascia over the tensor fascia latae is incised laterally, the interval between tensor fascia latae and sartorius is developed, the circumflex vessels are ligated or cauterized, the rectus femoris is retracted medially and the articular capsule is then incised. The surgery is performed using specific instrumentation for the minimally invasive approach designed by the implant’s manufacturer (Medacta Corporate, Strada Regina, Switzerland). Fluoroscopy is used intraoperatively to verify the inclination and version of the acetabular cup. Also, leg length is verified using the relationship between the lesser trochanter and the ischial tuberosity of the operated side and compared with the contralateral hip.

#### Posterolateral approach

The patient is positioned in lateral decubitus on a conventional operative table with lateral supports. The skin incision is positioned in the posterior 1/3 of the greater trochanter with 1/3 directed proximally toward the posterior superior iliac spine and 2/3 distally to the femoral diaphysis. The fascia over the gluteus maximus and the fascia latae are incised and retracted. The short external rotators are exposed and incised along with the articular capsule in one single flap. At the end of the procedure, the same flap is reattached to the greater trochanter trough osseus tunnels. Leg length is verified intraoperatively with the level of both knees.

For either approach, intraoperative stability maneuvers are performed with trial implants and after placing the final implants. The hip is flexed 90°, internally rotated 20°, adducted 15°, and in full extension is externally rotated for PL and combined flexion and external rotation for AMI.

### Preoperative protocol

All patients undergo the same preoperative protocol. They receive a kit with chlorhexidine soap and topic 2% mupirocin. Patients are instructed to shower with chlorhexidine soap the day before and on the day of the surgery. Topic mupirocin is applied in the nostrils, ears, and belly button every 12 h during 5 days before surgery. On the day of surgery, antibiotic prophylaxis consists of 2 g of cephazolin plus 1 g of vancomycin within 1 h of the incision. For patients allergic to penicillin, only 1 g of vancomycin is used. Additionally, antimicrobial incise drapes are used in every case. Furthermore, 1 g of tranexamic acid is applied intravenously at the time of the incision.

Patient older than 65 years are assessed preoperatively by internal medicine to optimize comorbidities such as diabetes and hypertension. Patients with HbA1c > 7% and Hb < 10 g/dL are postponed until correction is achieved.

### Postoperative protocol

The postoperative rehabilitation protocol is the same for both approaches. Patients are allowed to bear full weight with a walker in the immediate postoperative period, and no hip precautions are prescribed for either approach. Patients are discharged on day 1 if pain is well controlled and they tolerate ambulation. A booklet with self-directed mobility and strengthening exercises is provided to all patients. In the first visit at the clinic (day 12), pain control, wound healing, and adherence to pharmacological thromboprophylaxis are evaluated, and a prescription for outpatient physical therapy is also provided. Sutures are removed and education on the rehabilitation goals is strengthened. Then, patients are followed at 3, 6 and 12 months. The oxford hip score is applied in every visit. If a patient is not able to visit the clinic for follow-up, he is contacted by telephone.

### Statistical analysis

#### Exploratory analysis

Data distribution was evaluated using the Shapiro–Wilk test. Continuous variables are presented as mean and standard deviation if the distribution is normal, or as median and interquartile range (IQR) if the distribution is nonparametric. Categorical variables are presented as proportions.

#### Bivariate analysis

Comparison between continuous variables was performed using the Mann–Whitney *U* test if presented as medians or using the *T*-student test if presented as means. A Levene’s test for the analysis of variances was carried out before the T-student test. For paired data with normal distribution, a *T*-student test is performed, otherwise its nonparametric equivalent, the Wilcoxon test. Categorical variables are compared using the chi-square test or the Fisher’s exact test when the expected frequency is less than 5 cells. *p* values less than 0.05 were considered statistically significant.

### Ethical considerations

This study was approved by the Ethics Committee of our institution. This retrospective research is considered low risk.

## Results

A total of 244 patients were included in the analysis, where 124 underwent THA through the AMI approach. The median age was 62 years (IQR 50–72), 61% were females, the median BMI was 27.4 kg/m^2^ (IQR 23.9–30.5) and 81.7% were classified ASA II. The most frequent comorbidities were high blood pressure (75%) and diabetes mellitus (11.8%). The main indication for THA was primary hip osteoarthritis (91.5%). There were no significant differences in the distribution of demographic variables among groups (Table 1).

**Table 1:** Sociodemographic characteristics

	Surgical approach group		*p*
	AMI *n* = 124	PL *n* = 120	
Gender, *n* (%)			
Female	77 (62.1)	73 (60.8)	0.839
Male	47 (37.9)	47 (39.2)	0.839
Age, median (IQR)	62 (52–69)	63 (47–76)	0.527
BMI, median (IQR)	27 (23–30)	27 (24–30)	0.814
ASA, *n* (%)			
1	17 (13.7)	15 (12.5)	
2	95 (76.6)	88 (73.3)	
3	12 (9.7)	17 (9.7)	
Comorbidities, *n* (%)			
Hypertension	42 (33.8)	43 (36.4)	0.303
Diabetes	17 (13.7)	12 (10.7)	0.252
Smoking	17 (13.7)	12 (10.2)	0.623
Coronary disease	3 (2.4)	4 (3.2)	0.305
COPD	1 (0.8)	3 (2.5)	0.194
Surgical indication, n (%)			
Osteoarthrosis	111 (89.5)	94 (78.3)	
Avascular necrosis	10 (8.1)	15 (12.5)	
Inflammatory arthropathy	–	2 (1.6)	
Septic arthritis	–	2 (1.6)	
Hip dysplasia	2 (1.6)	2 (1.6)	
Post-traumatic arthrosis	1 (0.8)	5 (4.17)	

*AMI* anterior minimally invasive approach, *PL* posterolateral approach, *BMI* body mass index, *COPD* chronic obstructive pulmonary disease, *IQR* Interquartile range

**Fig. 1:**
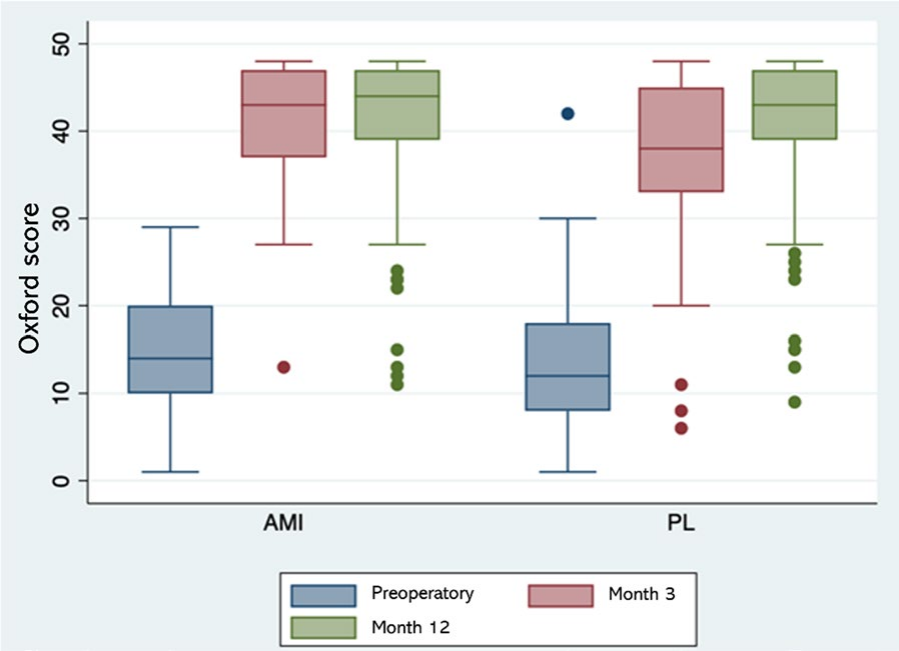
Comparison of functional outcomes with the oxford score

The preoperative functional status assessed with the Oxford Hip Score was not significantly different among groups. However, at the third postoperative month, superior outcomes were observed for AMI patients, with a median of 43 points (IQR 37–47) in contrast with 38 points (IQR 33–45) for the PL approach (*p* = 0.005). There were no differences at 12 months (Table 2, Fig. 1).

**Table 2:** Clinical outcomes reported with the oxford hip score

	Surgical approach group		*p*
	AMI *N* = 124	PL *N* = 120	
Oxford, median (IQR)			
Preoperatory	14 (10–20)	12 (8–18)	0.180
3 months POP	43 (37–47)	38 (33–45)	0.005
12 months POP	44 (39–47)	43 (38–47)	0.698

*AMI* anterior minimally invasive approach, *PL* posterolateral approach, *IQR* interquartile range, *POP* postoperative

**Fig. 2:**
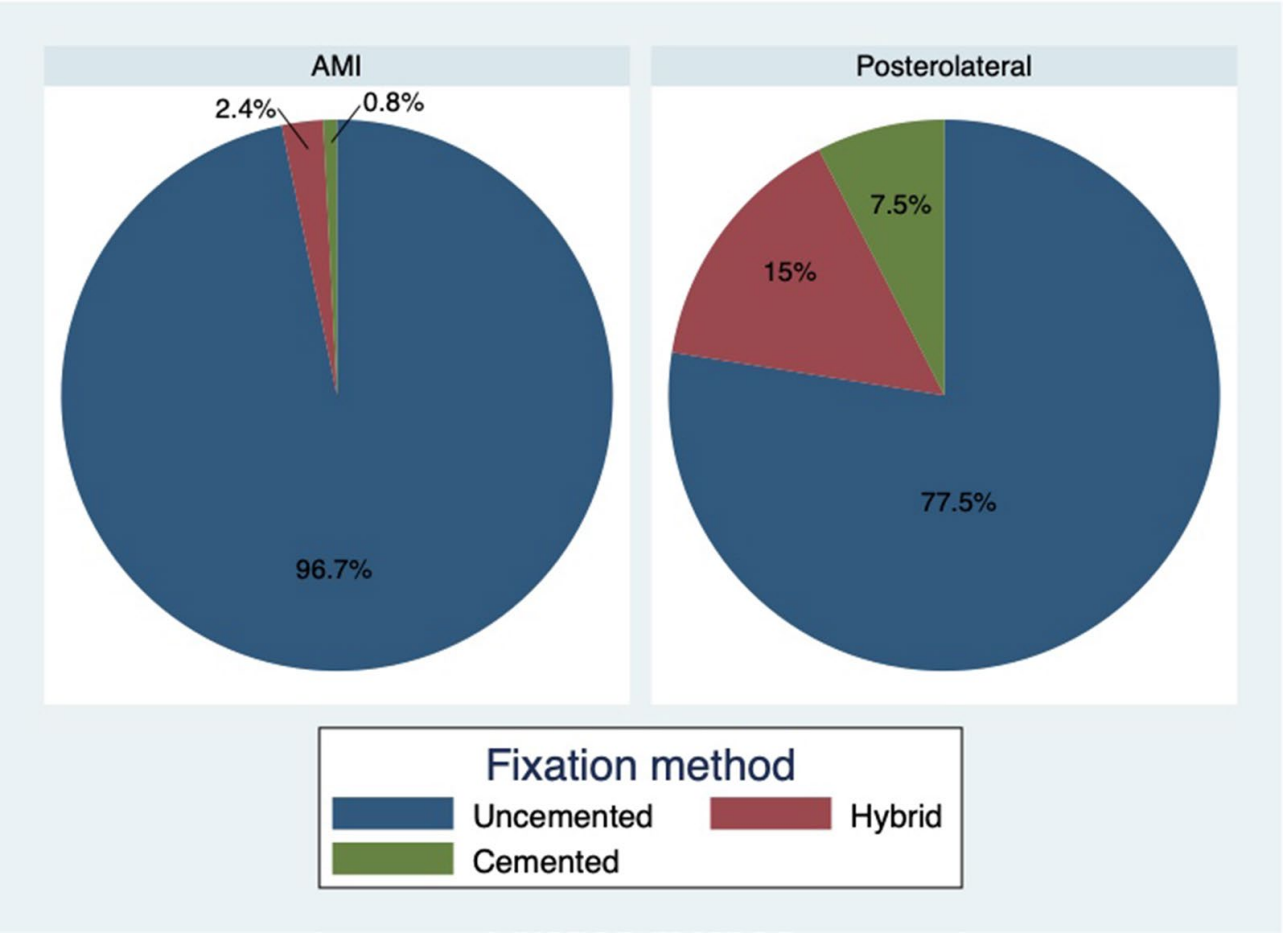
Hybrid, cemented and uncemented fixation distribution in both groups

Throughout the study, the median surgical time for the AMI approach was 123 min (IQR 108–145) and 83 min (IQR 68–95) for the PL approach, and this difference was statistically significant (*p* < 0.05). Hybrid, cemented and uncemented fixation were used (Fig. 2). Three cases of intraoperative trochanteric fractures were observed for the AMI approach and 1 case for the PL (p = 0.334). Postoperative hemoglobin levels were lower for the AMI group (10.3 g/dL vs. 11.1 g/dL, *p* = 0.082), there was a significant difference in postoperative hematocrit levels, but there were no significant differences in the transfusion rate. No differences in the length of stay were observed. (Table 3) No cases of postoperative sciatic nerve palsy were identified in either group. From the total of 18 lateral femorocutaneous nerve neuropraxias, 17 cases were in the AMI approach (Table 4).

**Table 3:** Surgical variables

	Surgical approach group		*p*
	AMI *N* = 124	PL *N* = 120	
Surgical time in minutes (IQR)	123 (108–145)	81 (68–95)	< 0.05
Intra operative complications (%)			
Greater trochanter fracture	3 (2.4)	1 (0.8)	0.334
Hospital length of stay in days (IQR)	2 (1–2)	2 (2–3)	0.164
Hemoglobin (IQR)			
Preoperative	13.6 (12.6–14.5)	13.4 (12.6–14.6)	0.895
Postoperative	10.3 (9.5–11.4)	11.1 (9.9–12.1)	0.082
Hemoglobin loss ≥ 2 g/dL, n (%)	90 (78.9)	75 (68.8)	0.084
Hematocrite, *n* (%)			
Preoperative	41.5 (38.1–43.7)	40.6 (38.8–43.2)	0.928
Postoperative	31.2 (28.6–34.6)	33.4 (30.2–36.4)	0.022
Transfusion requirement, n (%)	8 (6.5)	8 (6.7)	0.996

*AMI* anterior minimally invasive approach, *PL* posterolateral approach, *IQR* interquartile range

**Table 4:** Postoperative complications

	Surgical approach group		*p*
	AMI *N* = 124	PL *N* = 120	
Nerve palsy			
Sciatic	0	0	–
Lateral femorocutaneous	17 (13.7)	1 (0.83)	0.000
Dislocation, *n* (%)	0	5 (4.2)	0.022
Revision surgery, *n* (%)	2 (1.6)	3 (2.48)	0.632
Revision surgery indication, *n* (%)			
Dislocation	0	3 (2.28)	
Malalignment	1 (0.80)	0	
Periprosthetic fracture	1 (0.80)	0	
Surgical site infection, *n* (%)	5 (4)	4 (3.4)	0.572
Superficial, *n* (%)	3 (2.4)	3 (2.5)	0.959
Deep, *n* (%)	2 (1.6)	1 (0.8)	0.586
Thromboembolic disease, *n* (%)	0	2 (1.6)	0.208
1-year mortality, *n* (%)	0	6 (5)	0.023

*AMI* anterior minimally invasive approach, *PL* posterolateral approach

Five patients in the PL approach presented prosthetic dislocation, while no cases were identified in the AMI group (p = 0.022). No dual mobility acetabular implants were used. Of the former, 3 patients required revision surgery due to recurrent dislocation. One patient of the AMI approach presented early loosening of the acetabular cup, also requiring revision surgery. Nine surgical site infections were identified: 5 in the AMI group and 4 in the PL group; of these 6 were superficial and 3 deep infections. Deep infections were diagnosed according to the 2018 point-based definition published by Parvizi et al. [22] using both synovial and serum markers and confirmed by intraoperative cultures. All three cases of periprosthetic infection were managed with debridement, irrigation, prosthesis retention and 6 weeks course of antibiotics. Superficial infections were included cases presenting with erythema, purulent discharge or wound dehiscence without serum or synovial inflammatory markers elevation and were managed with oral antibiotics only. Two PL patients present pulmonary embolism in the immediate postoperative period (1.6%). Between 2017 and 2022, 6 deaths were registered for the PL approach; nevertheless, the cause of death was not related to the procedure (Table 4).

**Fig. 3:**
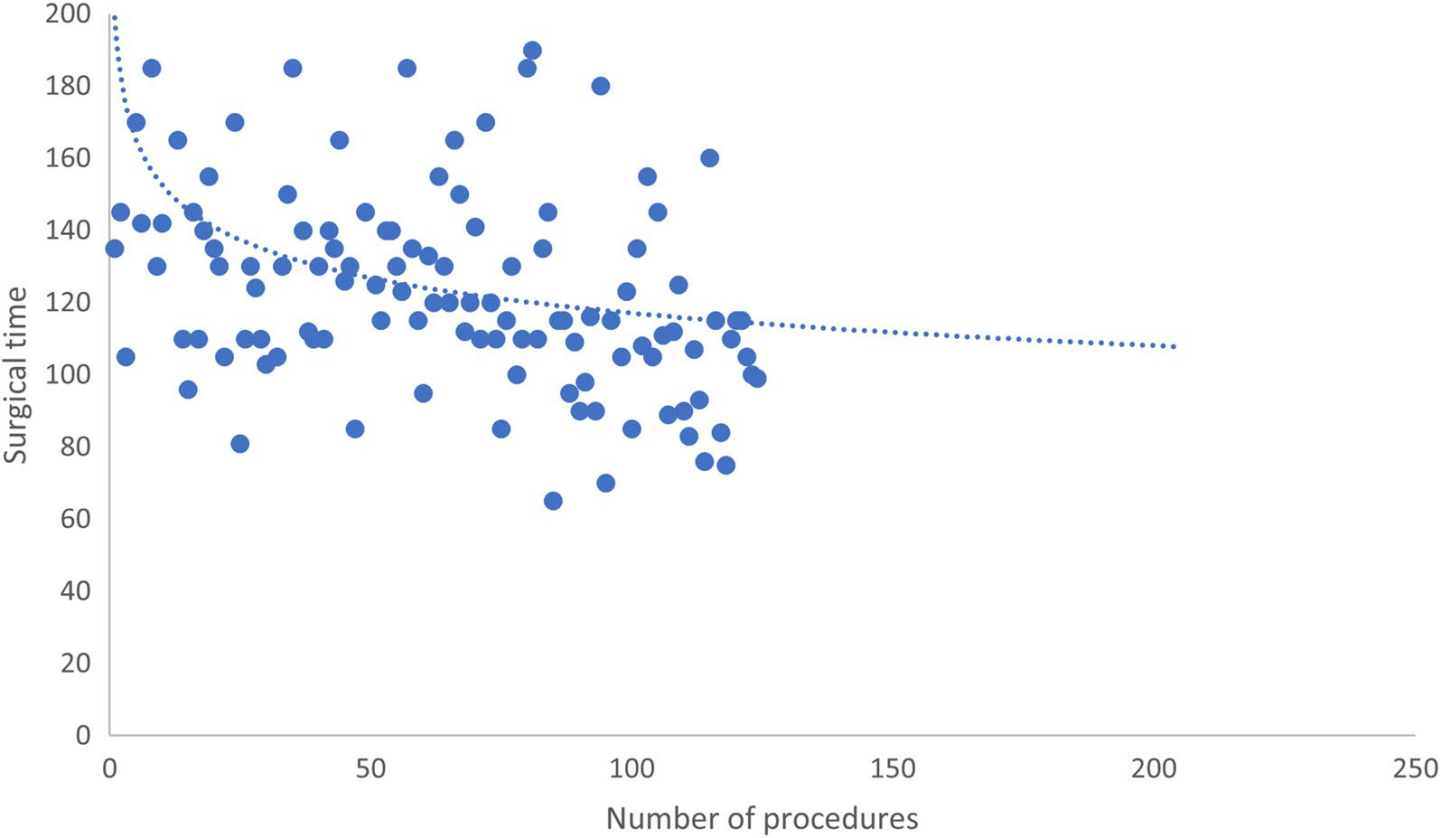
Learning curve in AMI approach, surgical time according to procedure number

The surgeon’s learning curve was defined as the decrease in surgical time as the number of cases performed increased. According to the date of the procedure, five groups of 25 consecutive patients were defined. Thus, the curve was obtained by plotting surgical time by the case number. A trend in the decrease in the surgical time along with the increase in the number of procedures performed was observed (Table 5, Fig. 3).

**Table 5:** Sub-analysis of surgical time and hospital length of stay in AMI group

Number of procedure*	01–25	26–50	51–75	76–100	101–125	*p*
Surgical time, median (IQR**)	142 (130–170)	130 (110–157)	125 (115–140)	115 (96–132)	107 (90–115)	0.000
Hospital length of stay, median (IQR**)	2 (2–4)	2 (2–3)	1 (1–2)	1 (1–2)	1 (1–1)	0.000

*Procedures were chronologically organized, then divided in groups of 25 to create the groups used for this sub-analysis

**IQR interquartile range

## Discussion

In the present study, the outcomes of the first 124 consecutive cases of THA through the AMI approach were compared with 120 cases through PL approach performed by the same surgeon at the same period. During this transition, the rate of dislocation and 1-year mortality was significantly lower for AMI patients; however, the proportion of other complications was not statistically significant between the two cohorts (Tables 3 and 4).

Moreover, functional outcomes (Oxford Hip Score) at 3 months after the index procedure were significantly better for the AMI approach compared to the PL approach (Table 2). According to the interpretation proposed by Nilsdotter et al., the results for AMI patients were *excellent*, while the results for the PL approach were *good* [23]. Similar findings have been reported in the literature, where faster recovery and better performance on functional and PROM scales are described in THA through AMI approach [24–26].

One of the main advantages attributed to the AMI approach is the lower risk of dislocation given that it is developed in an intermuscular plane, and it preserves the posterior hip soft tissues [27, 28]. We found a dislocation rate of 0% for the AMI approach compared to 4.2% for the PL approach (*p* = 0.022). This lower rate of prosthetic dislocation has been demonstrated in different randomized studies [9, 25, 26, 29], presented similar results in the long-term [29, 30] and decreasing the rate of revision surgery for instability [31].

Barrett and colleagues described an increased risk of intraoperative bleeding for the AMI approach compared to the posterolateral approach [25]. In our study, both cohorts had similar preoperative hemoglobin and hematocrit levels; however, these levels were lower for the AMI group on the first postoperative day (Table 3). A higher proportion of these patients had a decrease in hemoglobin ≥ 2 g/dL, a result of increase intraoperative bleeding, despite the systematic use of tranexamic acid. However, these results were not evidenced in the number of patients in the AMI group that required blood transfusions (6.5% [AMI] vs. 6.7% [PL], *p* = 0.996). Likewise, more cases of surgical site infections were identified in the AMI group, without a statistically significant difference in the proportion of this complication (4% [AMI] vs. 3.4% [PL], *p* = 0.572) or in the type of infection (Table 4). None of the cases was treated with revision surgery. These results are similar to those published by Aggarwal et al. in a retrospective study [13]. However, in randomized studies, no differences have been found in the prevalence of these complications [9, 25, 26, 29]. It is possible that our results may be related to the expected increased surgical time during the adoption of a new technique [9, 21, 29, 32].

We found that the surgical time was significantly longer for the AMI approach, with a median difference of 42 min (123 min [AMI] vs. 81 min [PL]; *p* < 0.05). Cheng et al. obtained a difference of 25 min, with a median of 100 min for the PL approach [29]. Gulbrandsen et al. found a difference of 12 min, with an average of 99 min for the PL approach [32]. Conversely, Zhao et al. reported a difference of 18 min, with an average time of 65 min for the posterolateral approach [9]. The time difference found in this study might be explained by the inclusion of all patients under the surgeon’s learning curve, unlike the study published by Graves et al., in which the cases completed during the first year of implementation of the AMI approach were excluded from the analysis [33]. The 124 patients reported in this study were the first patients undergoing THA with this approach, while in previous studies, treating surgeons already had experience with it. Nevertheless, the median time for the last 25 surgeries with the AMI approach was 107 min (Table 5), reducing the difference to 26 min. Similarly, after the 50th surgery with the AMI approach, the length of hospital stay decreased to 1 day. This finding might be related indirectly by less postoperative pain, shorter surgical time, and possibly less bleeding. Gulbrandsen et al. found that surgical time and length of hospital stay also decreased after a learning curve of 50 surgeries with the anterior approach [32]. Studies investigating the learning curve for the anterior approach conclude that around 50 procedures are needed to reach a plateau [16, 34].

Regarding intraoperative complications, there was a higher frequency of trochanteric fractures with the AMI approach, and similar findings have been described in the literature [9, 35, 36]. A possible explanation for the increased number of fractures with this approach is the challenging femoral exposure for canal preparation compared to the posterolateral approach, resulting in greater difficulty in broaching [5].

The main strength of this study is the direct comparison of the results from two different approaches performed by the same surgeon. This allows the description the surgeon's learning curve, the outcomes of these first 124 patients and compare them with what has been described in the literature. Nevertheless, this study presents some limitations. First, the retrospective design does not allow for randomization of patients; however, the sociodemographic variables were similar in both groups. Second, the number of patients included in each cohort may limit the power to quantify differences in rare outcomes, and the limited follow-up does not allow the evaluation of other relevant outcomes such as revision surgery. Third, we were not able to assess postoperative pain in an analogous scale due to a systematic error found during the review of medical records, and this outcome is another differentiating factor between both approaches. Lastly, the surgeon had extensive experience in the posterolateral approach and limited experience in the AMI approach, which could indicate a selection bias by selecting less complicated and low BMI cases for the AMI approach and vice versa, although the indications for surgery in both groups were similar.

## Conclusion

The anterior minimally invasive approach for total hip replacement is a good alternative for patients, offering better early functional outcomes and a lower dislocation rate, although it presents with longer surgical time and higher intraoperative blood loss during the learning curve. This study demonstrates that responsible innovation and safe implementation of new techniques for the benefit of patients is possible.

## Data Availability

The datasets used and/or analyzed during the current study are available from the corresponding author on reasonable request.
